# Supplementary figures and images for: Prevalence and distribution of Plasmodium vivax Duffy Binding Protein gene duplications in Sudan
Source: PLoS One. 2023 Jul 20;18(7):e0287668. doi: 10.1371/journal.pone.0287668 (PMC10358875; doi:10.1371/journal.pone.0287668)

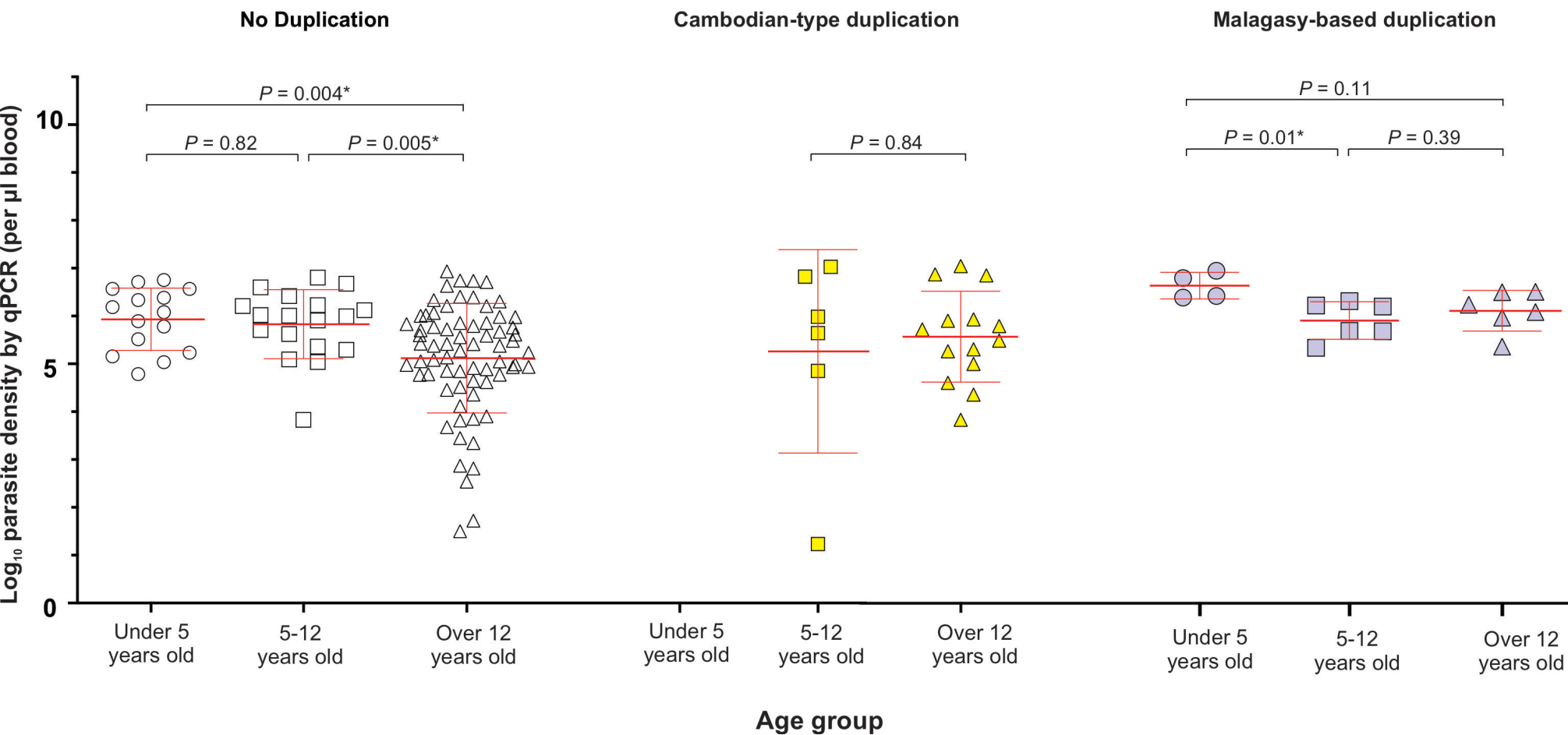

Supplement: S1 Fig — (PDF) [file pone.0287668.s003.pdf]
