# Supplementary material for: Prevalence and distribution of Plasmodium vivax Duffy Binding Protein gene duplications in Sudan
Source: PLoS One. 2023 Jul 20;18(7):e0287668. doi: 10.1371/journal.pone.0287668 (PMC10358875; doi:10.1371/journal.pone.0287668)
Supplement: S2 Fig — (A) A 613bp band observed in samples 124, 162, 167 and 168 using the duplication primer BF/AR for Malagasy-type duplication and a 736bp band in samples 18, 55, 99, 133 and 143 using primer BF/AR2 for Cambodian-type duplication. No bands were shown in the negative controls. (B) A ~650bp band observed in all samples using the controls primers AF/AR and AF2/AR2. No bands were shown in the negative controls. (C) A 650bp band observed in all samples using the controls primer BF/BR. No bands were shown in the negative controls. (PDF) [file pone.0287668.s004.pdf]

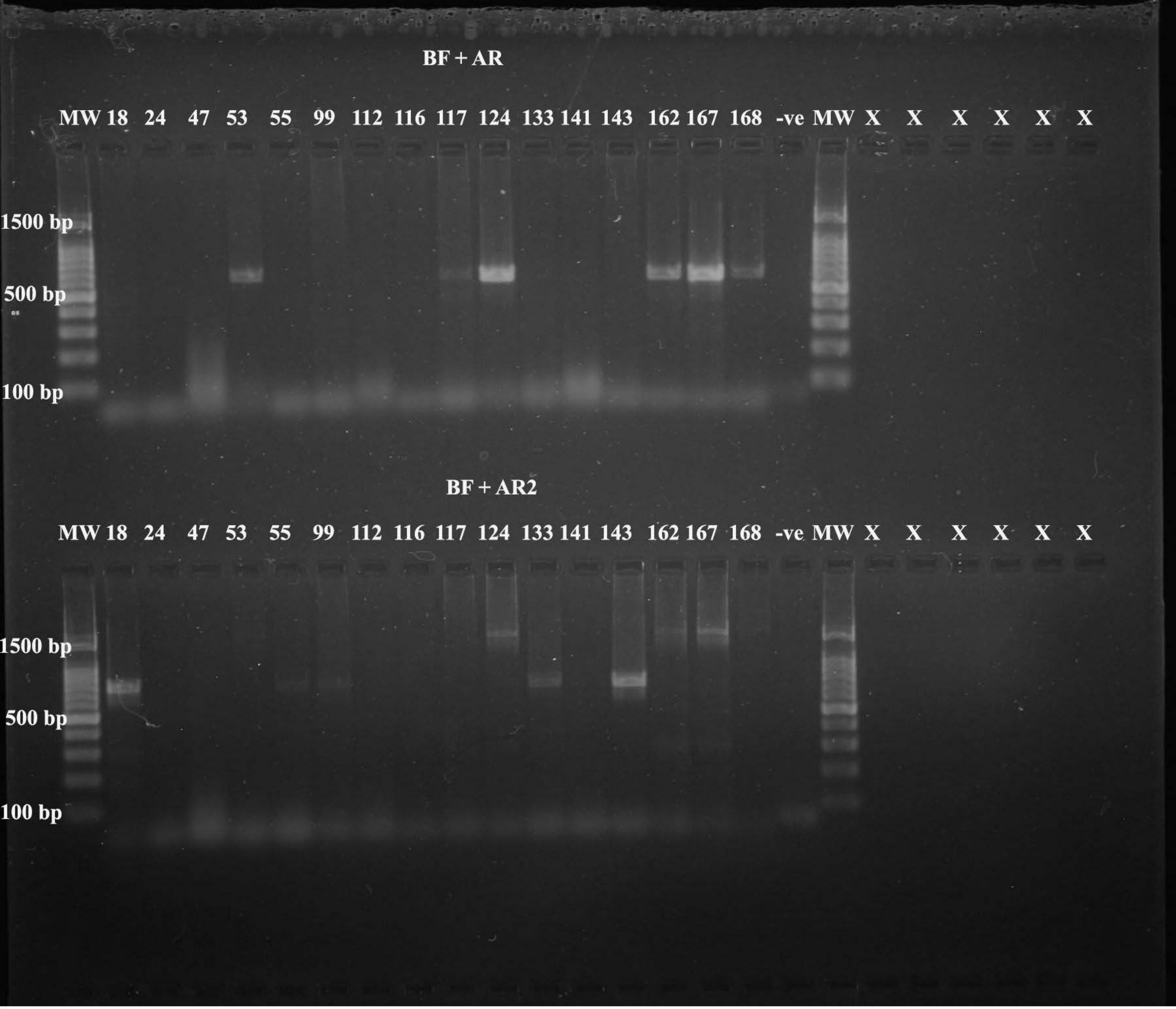

Suppl Figure 2 (A): Gel identification of *PvDBP* duplication types of 7 *P. vivax*-infected samples which have discrepancy result between PCR and qPCR using the duplication primer BF/AR for Malagasy-type duplication with 613bp band in samples 124, 162, 167 and 168 and primer BF/AR2 for Cambodian-type duplication with 736bp band in samples 18, 55, 99, 133 and 143 while no bands in the negative controls.

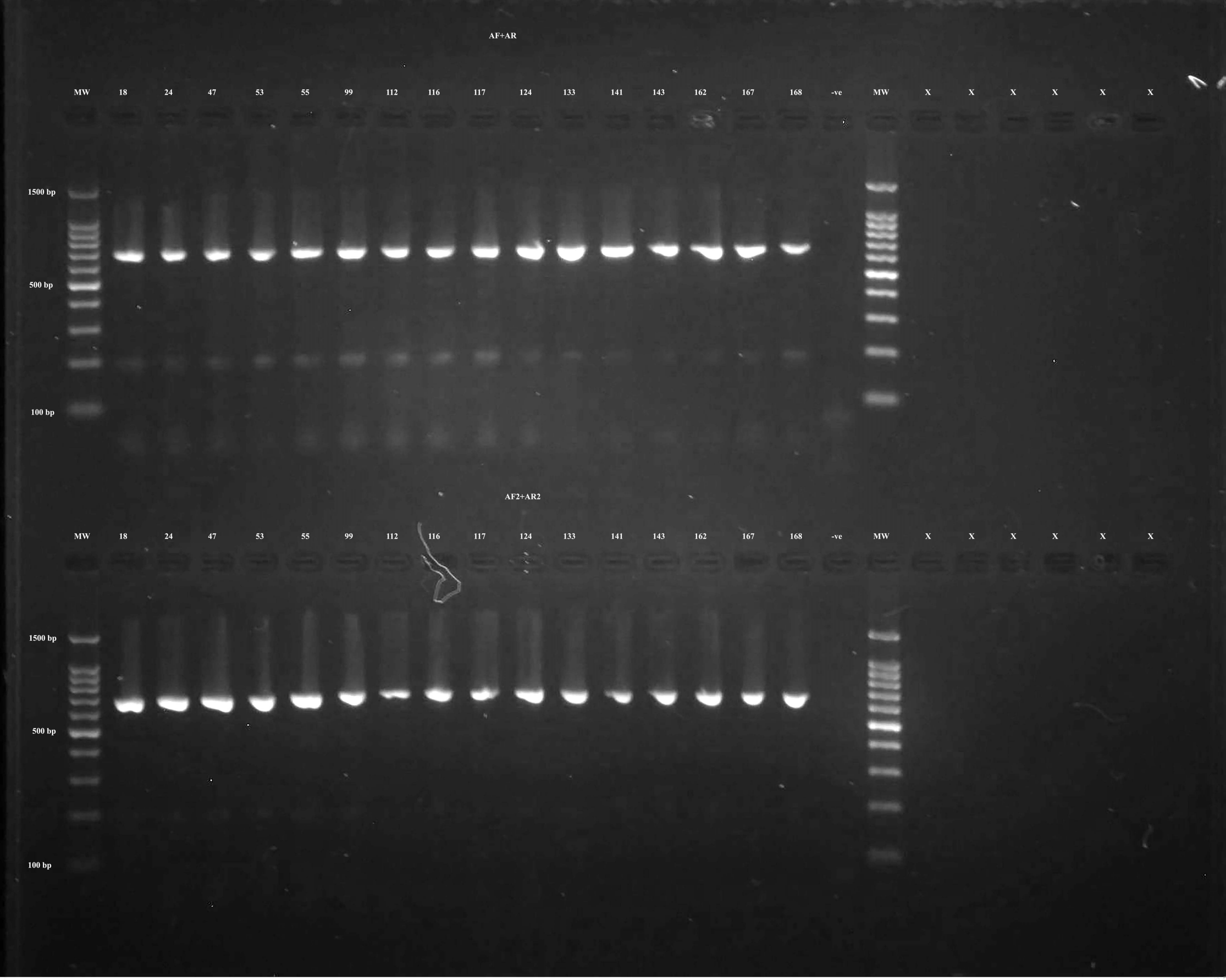

Suppl Figure 2 (B): Gel identification of *PvDBP* duplication types of 7 *P. vivax*-infected samples which have discrepancy result between PCR and qPCR using the controls primer (AF/AR, and AF2/AR2) showed approximately 650bp band in all samples, while no bands in the negative controls.

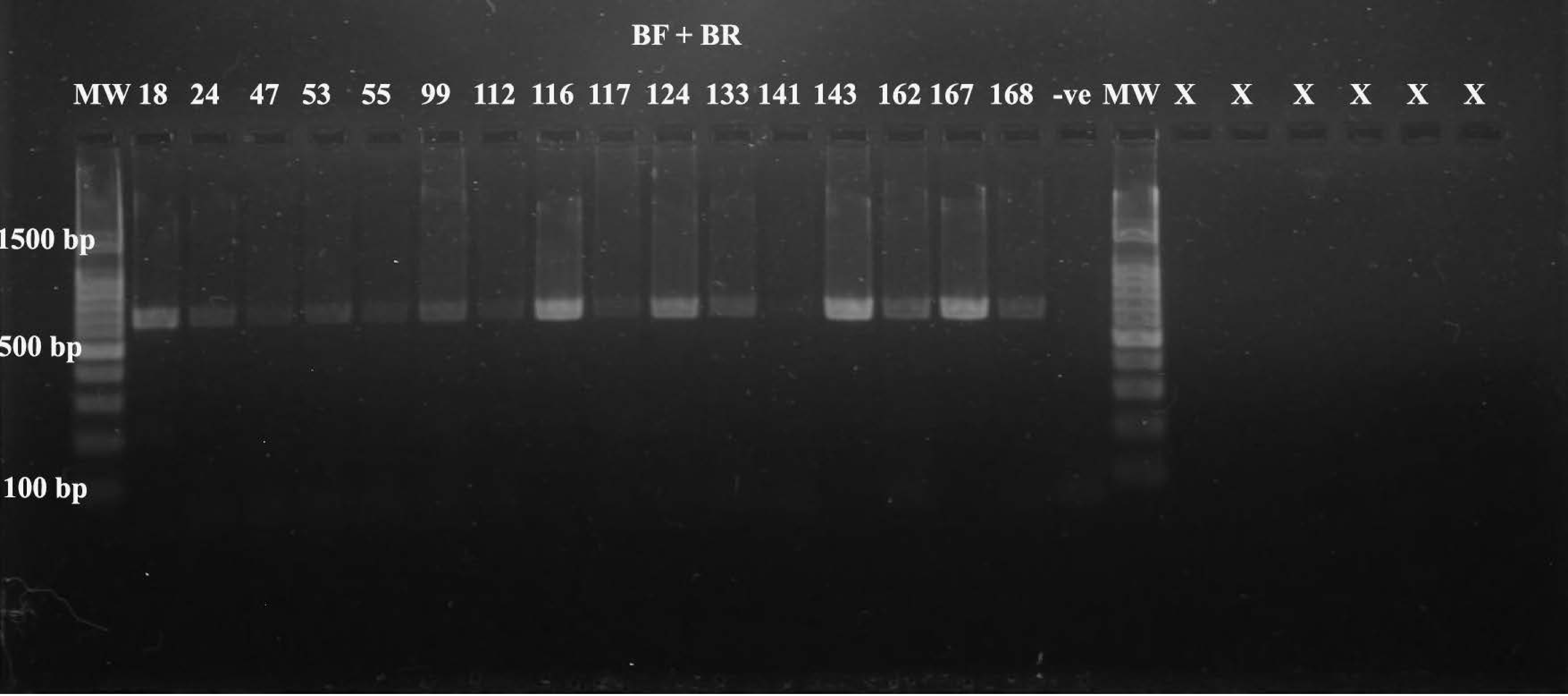

Suppl Figure 2 (C): Gel identification of *PvDBP* duplication types of 7 *P. vivax*-infected samples which have discrepancy result between PCR and qPCR using the control primer BF/BR showed approximately 650bp band in all samples, while no band in the negative control.
