# Supplementary material for: Prevalence and distribution of Plasmodium vivax Duffy Binding Protein gene duplications in Sudan
Source: PLoS One. 2023 Jul 20;18(7):e0287668. doi: 10.1371/journal.pone.0287668 (PMC10358875; doi:10.1371/journal.pone.0287668)
Supplement: Attachment — Submitted filename: Response to Reviewers.docx [file pone.0287668.s006.docx]

February 15, 2023

Dear Editor,

We submit the revised version of the manuscript entitled “Prevalence and distribution of Plasmodium vivax Duffy Binding Protein gene duplications in Sudan”. We are thankful to the reviewers’ constructive comments/suggestions and have taken all comments to improve this manuscript. All gel image data are provided as Supporting Information. We believe that this manuscript is scientifically valid and technically sound. In this revised version, we address the reviewers’ queries point-by-point. All changes can be viewed by track-changes in the uploaded word file. Our responses to the reviewers’ comments are detailed below in this response letter (reviewers’ comments in italic and our responses in bold).

We sincerely look forward to receiving your decision on this revised manuscript.

Yours Sincerely,

Safaa Ahmed

Editor: Comments/Suggestions

*1. Please ensure that your manuscript meets PLOS ONE's style requirements, including those for file naming. The PLOS ONE style templates can be found at*

*https://journals.plos.org/plosone/s/file?id=wjVg/PLOSOne_formatting_sample_main_body.pdf and*

*https://journals.plos.org/plosone/s/file?id=ba62/PLOSOne_formatting_sample_title_authors_affiliations.pdf*

**Our Response: We have revised the manuscript following PLOS ONE requirements.**

*2.Thank you for stating the following in your Competing Interests section:*

*"Authors declare that no competing interests exist."*

*Please complete your Competing Interests on* *the online submission form to state any Competing Interests. If you have no competing interests, please state ""The authors have declared that no competing interests exist."", as detailed online in our guide for authors at http://journals.plos.org/plosone/s/submit-now*

*This information should be included in your cover letter; we will change the online submission form on your behalf.*

**Our** **Response: We have inserted the no-conflict statement towards the end of the manuscript and in the online submission form.**

*3. PLOS ONE now requires that authors* *provide the original uncropped and unadjusted images underlying all blot or* *gel results reported in a submission’s figures or Supporting Information files. This policy and the journal’s other requirements for blot/gel reporting and figure preparation are described in detail at https://journals.plos.org/plosone/s/figures#loc-blot-and-gel-reporting-requirements and https://journals.plos.org/plosone/s/figures#loc-preparing-figures-from-image-files. When you submit your revised manuscript, please ensure that your figures adhere fully to these guidelines and provide the original underlying images for all blot or gel data reported in your submission. See the following link for instructions on providing the original image data: https://journals.plos.org/plosone/s/figures#loc-original-images-for-blots-and-gels.*

*In your cover letter, please note whether your blot/gel image data are in Supporting Information or posted at a public data repository, provide the repository URL if relevant, and provide specific details as to which raw blot/gel images, if any, are not available. Email us at plosone@plos.org if you have any questions.*

**Our Response: We have provided the original uncropped and unadjusted gel images as Supporting Information in S1_raw_images.**

*4. Please* *include your full ethics statement in the ‘Methods’ section of your manuscript file. In your statement, please include the full name of the IRB or ethics committee who approved or waived your study, as well as whether or not you obtained informed written or verbal consent. If consent was waived for your study, please include this information in your statement as well.*

**Our Response: The full ethics statement is now inserted in the Material & Method section.**

*5. We note that Figure 3 in your submission contain map/satellite image which may be copyrighted. All PLOS content is published under the Creative Commons Attribution* *License (CC BY 4.0), which means that the manuscript, images, and Supporting Information files will be freely available online, and any third party is permitted to access, download, copy, distribute, and use these materials in any way, even commercially, with proper attribution. For these reasons, we cannot publish previously copyrighted maps or satellite images created using proprietary data, such as Google software (Google Maps, Street View, and Earth). For more information, see our copyright guidelines: http://journals.plos.org/plosone/s/licenses-and-copyright.*

**Our Response: The License (CC BY 4.0) is provided in online submission.**

Reviewer #1:  Comments/Suggestions

*1. The main concern of this manuscript, as also stated by the authors, is the high* *discrepancy rate of PvDBP copy number determination between quantitative real time PCR and genotype-specific PCR (16 discrepancy samples out of 43 concordant samples). It is unclear which results (qPCR vs genotype-specific PCR) were used for subsequent analysis. With small sample size in this study, such discrepancy and potentially incorrect determination of copy number variation in this locus may preclude meaningful statistical analysis.*

**Our Response: We used** **genotype-specific PCR for all subsequent analysis as stated in the result, especially for Figure 3 qPCR was used (P11 to 16. L229-309). We have repeated PCR and qPCR for 16 samples twice and confirm that 7 out of 43 samples showed discrepancy results. We provide the genotype-specific PCR gel images and qPCR data in NEW Supplementary File 2.**

*2. Please include the range of parasitemia and standard deviation of the average parasitemia (line 216). The mean parasitemia determined by qPCR would be approximately equivalent to 0.001% by microscopy detection which is around the microscopy detection limit. Did* *most patients have submicroscopic parasitemia? In P. vivax endemic areas outside Africa, most infected individuals have symptomatic malaria. Please* *describe* *the clinical status of the patients recruited in this study and parasitemia status of vivax malaria in Africa and outside Africa since parasitemia is an important parameter in this study.*

**Our Response: Yes, most patients have microscopic parasitemia, and the range, mean and standard deviation of** **microscopic parasitemia have been added. The clinical symptoms of the patients are also described (P11; L221-224).**

*3. There is no sufficient statistical power to draw a conclusion for the Duffy-negatives (n = 3) (lines 228-230).*

**Our Response: Because only 3 Duffy-negatives (C/C) were included in this study, the sample size is too small for any statistical testing. We are currently expanding Duffy-negative samples for DBP copy number assays with the goal to draw confident comparisons with Duffy-positive ones as described in this study (P19 L373-375).**

*4. It is confusing that the magnitudes of parasitemia for Malagasy-type and Cambidian-type bearing parasites are not significantly different while only parasites with the former genotype has significantly higher parasitemia than those having single copy of PvDBP. Please explain.*

**Our Response: We stratify the parasitemia of infections with single, Cambodian, and Malagasy copies by age groups. Among all comparisons, only infections with the Malagasy duplications have significantly higher parasitemia than single-copy ones in age under 5 and over 12 years old (Supplementary Figure 1; P14 L284). Limited samples with the Cambodian and Malagasy duplications may explain such differences.**

*5. In this study, individuals above 12 years old showed a significantly lower parasitemia than those under 5 years old as well as those aged 5-12 (lines 218-220). Please verify that the analysis of parasitemia among patients infected with single-copy, Malagasy-type and Cambodian-type parasites is not affected by the age of the patients.*

**Our Response: We have addressed this comment by stratifying the parasitemia analyses by age group as mentioned in the above response.**

*6. Line 87, increased mRNA levels per se may not always directly indicate protein expression. Please revise.*

**Our Response: We agree with this comment, and thus we stated that ‘Increased *PvDBP* copy number may lead to increased mRNA levels’. We also add reference #23: Popovici et al. Nat Commun. 2020;11(1):953 to support this statement.**

*7. The* *references need some attention regarding the format and upper-case/lower-case letters.*

**Our Response: All references are revised.**

Reviewer #2: 

Major comments:

*1. Provide a* *brief description about malaria incidence in the areas of study, because this information is relevant to understanding of PvDBP duplication prevalence in the different areas.*

**Our Response: We have included a brief description on malaria incidence in the study areas in the M&M (P5 L106)**

*2. Line 196. There is an error in the formula presented. The delta CT should be calculated by: ΔCT = CT target – CT reference (Livak & Schmittgen 2001, METHODS 25, 402–408). Then, the delta, delta CT is calculated by ΔΔCT = ΔCT test sample – ΔCT calibrator sample. Please make sure that it is a typo and not an error in the formula used to estimate copy number of pvdbp.*

**Our Response: We have cited three references for the calculation of copy number (refs# 6,29 and 30 and checked the accuracy of the equations**.

*3. Line 243. The authors claim that the parasitemia was significantly higher in samples with the Malagasy-type duplication compared to those without duplication. Have you checked if the age (i. e. confounding factor) could explain this difference since parasitemia is significantly different among age groups? This analysis will be important to support the manuscript´s main finding of higher parasitemia in infections with the Malagasy-type duplication.*

**Our Response: We stratify the parasitemia of infections with single, Cambodian, and Malagasy copies by age groups. Among all comparisons, only infections with the Malagasy duplications have significantly higher parasitemia than single-copy ones in age under 5 and over 12 years old (Supplementary Figure 1; P14 L284). Limited samples with the Cambodian and Malagasy duplications may explain such differences.**

*4. Lines 325-327. This is contrary to the result that has been shown in Lines 247-249 (“The frequency of PvDBP duplication was not significantly different between heterozygous Duffy positives (C/T; 36/127=28.3%) and homozygous Duffy-positive (T/T; 5/15=33.3%) individuals (p-value=0.19; Table 2), …”). Please clarify this part of the Conclusion session.*

**Our Response: We have clarified that the frequency of PvDBP duplication was not significantly different between heterozygous Duffy positives (C/T) and homozygous Duffy-positive (T/T) in the Discussion (P19 L381-384).**
