# Supplementary material for: Prevalence and distribution of Plasmodium vivax Duffy Binding Protein gene duplications in Sudan
Source: PLoS One. 2023 Jul 20;18(7):e0287668. doi: 10.1371/journal.pone.0287668 (PMC10358875; doi:10.1371/journal.pone.0287668)
Supplement: Attachment — Submitted filename: Response letter.docx [file pone.0287668.s007.docx]

*Journal Requirements:

Please review your reference list to ensure that it is complete and correct. If you have cited papers that have been retracted, please include the rationale for doing so in the manuscript text, or remove these references and replace them with relevant current references. Any changes to the reference list should be mentioned in the rebuttal letter that accompanies your revised manuscript. If you need to cite a retracted article, indicate the article’s retracted status in the References list and also include a citation and full reference for the retraction notice.*

**Our Response:**

**All references are checked and revised. Reference 32 was mistakenly deleted in the last submitted version due to sentence rearrangement but has now inserted back in this revision***.*

*Reviewer #2: Additional comments

1. The paper requires revision, both in content and language. Results are frequently reported in a confused way. For example, in “Frequency of PvDBP duplications among Duffy groups…” the initial description of PCR results (gels, etc) was presented after showing complete data in Table 2.*

**Our Response:**

**We have carefully checked and modified the content and language throughtout the text in this version. We have made substantial reorganization of text, tables, and figures in the Results based on to the suggestion (P12-13).** *2. This reviewer could not find Suppl. Figure 1. Thus, I could not evaluate one of the points raised by this reviewer concerning age as a confounding factor.*

**Our Response:**

**Suppl. Figure 1** **was** **uploaded again in this revision**

*3. Indicate the statistical test performed for each analysis.*

**Our Response:**

**We have described in detail the statistical tests used for age, Duffy groups, and PvDBP duplication comparisons (P10 L202-210)***.*

*4. Please, replace “Multi-DBP” with multicopy DBP, “Single-DBP” to single copy DBP to become clear to the readers.*

**Our Response:**

**The words are now replaced in Tables 2 and 3.**

*5. Provide the relative frequencies for Table 2 and 3 to make it easier the comparison between groups.*

**Our Response:**

**All relative frequencies are now added in Tables 2 and 3.**

6. Suppl. Figure 2A presents an error in primers nomenclature.

**Our Response:**

**Primer nomenclature was corrected in Supplementary Figure 2A and 2B.**
